# Relationships between Parenting Skills and Early Childhood Development in Rural Households in Western China

**DOI:** 10.3390/ijerph17051506

**Published:** 2020-02-26

**Authors:** Jingdong Zhong, Yang He, Yuting Chen, Renfu Luo

**Affiliations:** 1 School of Economics, Peking University, Beijing 100871, China; jdzhong@pku.edu.cn; 2 China Center for Agricultural Policy, School of Advanced Agricultural Sciences, Peking University, Beijing 100871, China; heyang.ivy@pku.edu.cn (Y.H.); ytchen2017@nsd.pku.edu.cn (Y.C.)

**Keywords:** early childhood development, parenting skills, human capital, rural China

## Abstract

This paper empirically investigates the relationships between caregivers’ parenting skills and early cognitive, language, motor, and social-emotional development of children aged 6–24 months. The study is based on data from a survey conducted in 100 villages in a typical poor rural area in western China. A total of 1715 households were enrolled in the study. In the study, Parent and Family Adjustment Scales (PAFAS), Bayley Scales of Infant Development version III (BSID-III), and a socioeconomic questionnaire were used to measure caregiver’s parenting skills, child’s development outcomes, and socioeconomic characteristics in sample households, respectively. Multivariate regression was used to estimate the relationship between a caregiver’s parenting skills and the child’s development outcomes. The results show that, first, parenting skills are positively and significantly associated with children’s cognitive, language, motor, and social-emotional development, and the link between parenting skills and social-emotional development is the strongest; second, the correlation between parenting skills and development outcomes varies across socioeconomic characteristics and parenting skill dimensions. The results provide evidence for the relationship between parenting skills and early childhood development in rural households in western China. Our findings also suggest that interventions aimed at improving caregivers’ parenting skills during the early stages are necessary for human capital development in rural China.

## 1. Introduction

Developmental delay remains a significant problem among young children in rural areas of China. New estimates show that, in four major subpopulations of rural China, 85% of the children aged 0–3 years suffer from at least one kind of developmental delay [1]. Specifically, nearly half (49%) of the children exhibited cognitive delays, and 30% had motor delays, while more than half were delayed in language skills (52%) and social-emotional skills (53%) [1].

Such early delays are detrimental to adult outcomes. In theoretical analyses, a well-established framework shows that skills formed at an early stage can effectively boost the later skill development in the long-term [2,3]. Empirical evidence further proves that early childhood development (ECD) is critical to one’s physical health [4,5], income in the labor market [6,7], social mobility [8], and other welfare markers in adulthood [9].

Studies in developed countries have documented that parenting skills are important to ECD outcomes. To be specific, different styles of parenting skills bring different benefits to child development. Enriching and effective parent-child interactions, such as linguistic and cognitive stimulation, would result in positive outcomes in children’s learning and social–emotional development, as children were positively immersed in learning interactions to build their emerging skills with their responsive parents [10,11]. Children tend to increase their engagement with the environment when mothers learned to tune in to their child’s signals and needs [10]. The avoidance of negativity and intrusiveness, such as decreased spanking, controlling, or parental detachment behaviors of parents, were found to be associated with improvements in children’s academic and behavioral skills [11,12]. Greater lability in parental warmth was associated with a higher incidence of internalizing problems and a lower social competence of the child [13]. However, these studies are all conducted in developed countries. Since the giant differences in environment and context, the findings in these studies can only provide limited information to rural households in western China.

Existing studies in China have focused on the link between poor ECD outcomes and poor parenting practices in rural areas. Only 12.6% of rural caregivers had read with their child in the previous day, and less than 40% had played (39.2%) and sang songs (37.5%) with their child [14]. Similar results are found in the caregiver’s story-telling behavior—only 13.8% of caregivers had told stories to their child [15]. On average, rural children played alone for more than 2.5 hours per day, which indicates a lack of interactive parenting with these children, too [16]. In addition to parenting activities, rural households also owned less play materials, in both quantity and variety [17,18]. Poor parenting practices have been found to be highly correlated with childhood developmental delays [14,15,16,17,18]. However, to the best of our knowledge, few studies have investigated the relationships between parenting skills and ECD outcomes in rural China.

This paper has three specific objectives. First, this paper aims to explore the relationships between caregivers’ parenting skills and the child’s early cognitive, language, motor, and social-emotional development in Chinese rural households. Second, it aims to figure out whether socioeconomic characteristics affect the relationships between parenting skills and development outcomes. Third, it plans to examine the relationships between different styles of parenting skills and different development outcomes. 

To achieve this objective, we first proposed the following study hypotheses. 1. Caregivers’ parenting skills are positively associated with the child’s development outcomes in Chinese rural households. 2. Relationships between parenting skills and development outcomes vary across socioeconomic characteristics. 3. Different dimensions of parenting skills have different predictive abilities on child outcomes. Then, we conducted a large-scale survey in 100 villages in a typical poor rural area of western China, and collected a dataset containing 1715 child–caregiver pairs from representative rural households. In this dataset, early cognitive, language, motor, and social-emotional development of children aged 6–24 months and their caregiver’s parenting skills were well measured by internationally standardized psychological instruments.

This paper contributes to the existing literature in three aspects. First, this work found evidence that, in rural households, caregivers’ parenting skills are significantly and positively associated with child development in cognitive, language, motor, and social-emotional skills. Second, the findings reveal that the relationships between parenting skills and development outcomes vary across socioeconomic characteristics, and female children, children who have no siblings, and who live in non-dibao households benefit more from parenting skills. Third, the findings also reveal that different dimensions of parenting skills have heterogeneous predictive abilities on child outcomes. Low coercive parenting might be the most important factor for child development.

## 2. Methods

### 2.1. Sampling

The survey was conducted in 22 nationally-designated poverty counties in Southern Shaanxi Province, which is a typical poverty-stricken rural area. Shaanxi Province ranked in the bottom half among all provinces in terms of the per capita income in 2016, and over 40% of its population lived in rural areas.

The protocol to select the study sample was as follows. First, we obtained a comprehensive list of all towns in each county from the local regulatory authority. From the list of towns in the study counties, we randomly chose 100 towns. Second, within each town, we randomly selected one village to participate in the study. Finally, we obtained a list of registered births from the local official in each sample village. From the list, we identified all households with children aged 6–24 months, and included sample children and their caregivers in the survey. We ended up with 1720 households that met the criteria. After excluding five sample households with missing survey information, our final sample includes 1715 sample households from 100 villages. The average sampling error was used to measure the real sampling error. Cronbach’s alpha was used to measure the internal consistency reliability. 

### 2.2. Procedure

In 2016, we collected data on three types of information in each sample household—caregiver’s parenting skills, child’s development outcomes, and socioeconomic status. We used the following instruments in the survey.

(1) Parent and Family Adjustment Scales (PAFAS). We first identified the primary caregiver of each child as the individual most responsible for the child’s daily care. The primary caregiver was then administered a detailed questionnaire adapted from the PAFAS, which was designed by [19] to measure parenting skills. Previous studies have demonstrated the PAFAS to yield both reliability and validation in developed countries, such as Australia [19], and developing countries, such as Indonesia [20]. The PAFAS was adapted to the Chinese language and context in [21].

As presented in Table A1, the inventory of PAFAS has two components—one is the 18-item parenting scale and the other is the 12-item family adjustment scale. The parenting scale has four subscales—parental consistency (five items), coercive parenting (five items), positive encouragement (three items), and parent–child relationship (five items). The family adjustment scale has three subscales—parental adjustment (five items), family relationships (four items), and parental teamwork (three items). 

Twelve out of the 30 items of the PAFAS inventory are designed as reverse-scored (higher scores indicating higher dysfunction levels), while the remaining 18 are designed as positive-scored (higher scores indicating lower dysfunction levels). For the 12 reverse-scored items, we used three minus their original scores to keep consistency across all items, as the primary caregivers were asked to use the 4-point scales ranging from 0 (not true at all) to 3 (very much true) to score the items. The Cronbach’s alpha coefficient of the PAFAS is larger than 0.7, which demonstrated good internal consistency in our sample [22]. We summed up the scores of the 30 items to calculate the total score of PAFAS. A higher total score of PAFAS indicated better parenting skills.

(2) Bayley Scales of Infant Development version III (BSID-III). The child was administered the BSID-III test, which was designed by [23] as a standardized instrument to evaluate the developmental functioning of a child from birth to age 3 years [24]. The BSID-III includes four scales, assessing the child’s cognitive, language, motor, and social-emotional development, respectively. These scales have been formally adapted to the Chinese language and context, and have been used in multiple studies across rural China [1]. 

The BSID-III test was administered by trained enumerators using a standardized set of toys and a detailed scoring sheet. The caregiver of each child was present but was not allowed to assist the child during the test. All enumerators attended a week-long training course on how to administer the BSID-III before the survey, and they were blind to the study. 

The test takes into account each child’s age in months and whether he or she was premature at birth. These two factors are combined with the child’s performance on a series of tasks. The assessments of cognitive, language, and motor skills depend on scores that are given according to the child’s successful completion of the tasks [23], while the social-emotional score comes from caregivers’ responses to relevant questions developed from the Greenspan Social-Emotional Growth Chart [25]. Higher scores indicate better development of the child.

(3) Socioeconomic survey. The questionnaire was administered to the primary caregivers to collect data on the socioeconomic characteristics of each household, including the child’s gender, the child’s age in months, whether the child had siblings, the caregiver’s age, the caregiver’s year of schooling, and whether household receives dibao (a social security support program in China).

### 2.3. Descriptive Statistics

Table 1 presents the descriptive statistics for our sample. In terms of child development, the mean scores of cognitive, language, motor, and social-emotional scales in our sample are 96.00, 92.41, 97.21, and 85.94, respectively. The mean scores (standard deviation) among a healthy population are expected to be 105 (9.6), 109 (12.3), 107 (15), and 100 (16) for the cognitive scale [26], the language scale [27], the motor scale [28], and the social-emotional scale [23], respectively. That is, the mean scores of cognitive, language, social-emotional scales in our sample are about one deviation lower than the reference mean scores of healthy children.

In terms of socioeconomic characteristics, approximately half (52%) of the children were male; the children were slightly over 14 months old on average; approximately half (51%) of the children had siblings; the primary caregivers were slightly over 35 years old on average; the caregiver’s year of schooling was 8.07 on average; and 11% of households received social security support.

### 2.4. Statistical Analysis

In order to identify the association between caregivers’ parenting skills and the child’s development, we used the multivariate regression analysis as follows:

(1) $$bayley_{i}=\alpha +\beta_{1}pafas_{i}+\gamma X_{i}+u_{j}+\varepsilon_{i}$$

where $bayley_{i}$ is the child *i*’s cognitive, language, motor, and social-emotional score in the BSID-III; $pafas_{i}$ is the caregiver *i*’s PAFAS total score; $X_{i}$ are covariates on socioeconomic characteristics, including the child’s gender, the child’s age in months, whether the child has siblings, the caregiver’s age, the caregiver’s year of schooling, and whether the household receives dibao; $u_{j}$ are the village fixed effects to control for the unobserved heterogeneity at the village level; $\varepsilon_{i}$ is the random error term. We adjusted the standard errors to account for clustering at the village level. The Stata 15.0 was used for data analysis. 

## 3. Results

### 3.1. The Relationship between Parenting Skills and Development Outcomes

Table 2 reports the ordinary least squares (OLS) estimates of Equation (1). Parenting skills are positively associated with the child’s cognitive and motor development at the 5% significance level, and are positively associated with the child’s language and social-emotional development at the 1% significance level. In terms of magnitude, a one standard deviation (SD) increase in parenting skills was accompanied by a 0.05, 0.09, 0.05, and 0.24 SD improvement in the child’s cognitive, language, motor, and social-emotional development. This indicates that the correlation between the caregiver’s parenting skills and the child’s social-emotional development is strongest in rural households in western China.

In contrast with cognitive skills, social-emotional skills have more lasting effects on the child’s adult welfare [29]. Social-emotional skills not only have greater malleability than cognitive skills [30], but could also improve the child’s future cognitive skills, as measured by test scores [3,31]. The results in Table 2 suggest that interventions aimed at the caregiver’s parenting skills might be effective to boost the child’s development outcomes.

### 3.2. Heterogeneity

Table 3 reports the OLS estimates of Equation (1) by subsamples. Panel A shows that parenting skills are positively and significantly associated with the cognitive development of a child who is female (Column 2), lives in a nontdibao household (Column 4), and has no siblings (Column 6). The association between parenting skills and the child’s language development was not significant among dibao households only (Column 3, Panel B). Panel C shows a positive and significant association between parenting skills and the motor development of a child who is female (Column 2), lives in a nondibao household (Column 4), and has no siblings (Column 6), which is similar to the findings of cognitive development in Panel A. The relationships between parenting skills and the child’s social-emotional development were positive and significant in all subsamples, and no significant difference in magnitude were found among subsamples (Panel D).

In contrast with nondibao households, dibao households have a lower income, thus face tighter budget constraints, and might invest less in their child. This might restrain child development [32,33]. Similarly, having siblings might spread the parental investments, which in turn, may hinder child development [34]. In such households, only improving the parenting skills might not substantially foster the development outcomes that are highly correlated with parental investments.

Given the multifactorial measure of parenting skills, we then examined which subscale is most strongly correlated with the child’s neurodevelopment. We replaced the PAFAS total score with PAFAS seven subscale scores as independent variables in Equation (1), and report the OLS estimates in Table 4. Higher parental consistency was beneficial for the child’s cognitive development (Column 1). Lower coercive parenting and a better parent–child relationship could benefit the child’s language development (Column 2). Lower coercive parenting could also improve the child‘s motor development (Column 3). Four out of seven subscales, including coercive parenting, positive encouragement, parent–child relationship, and parental adjustment, were positive and significantly associated with child’s social-emotional development (Column 4).

These results suggest that, on the one hand, parenting skills are indeed strongly correlated with the child’s social-emotional development. On the other hand, low coercive parenting might be the most important factor for child development, followed by the parent–child relationship. This is informative for policy-makers to design targeted interventions.

## 4. Discussion

In this paper, we examined the relationship between parenting skills and early childhood development in rural households in western China. Using survey data collected from representative households in 100 villages in 2016, we applied multivariate analysis to investigate the statistical correlation between the caregiver’s parenting skills and the child’s cognitive, language, motor, and social-emotional development.

The study has the following findings. First, the full sample estimates reveal that, after controlling for the socioeconomic characteristics and village fixed effects, parenting skills are positively and significantly associated with the child’s neurodevelopment, particularly with the child’s social-emotional development. Second, subsample estimates show that female children, children who have no siblings, and who live in nondibao households benefit more from parenting skills. Third, different dimensions of parenting skills have heterogeneous predictive abilities on the child’s development outcomes. Low coercive parenting might be the most important factor for a child’s development, which is positively and significantly associated with the child’s language, motor, and social-emotional development.

Given the nature of social-emotional skills having greater malleability compared to cognitive skills [30], and that they exert more lasting effects on one’s lifetime outcomes [29], the findings of this paper show that the caregiver’s parenting skills during the early stages of childhood could be critical to his/her future achievements in the long term. Furthermore, the findings are also consistent with the evidence found by previous studies on the effects of family income [32,33] and children quantity [34] on the child’s development. 

In terms of family income, in the USA, the increase in income raises school-aged children’s combined math and reading test scores [32]. That is, lower income in the household might restrain child development [33]. Our findings suggest that, in rural China, the link between the caregiver’s parenting skills and the child’s early development outcomes is hampered in the dibao households, which have a low income and receive social security support from the government. In such households, disfunction of parenting skills could be accompanied by the child’s early cognitive delays. For those children, early cognitive delays would further restrain their academic achievements in school [35].

In terms of children quantity, in Chinese rural households, having siblings exerts negative and significant impacts on a child’s early development, by spreading the parental investments among children [34]. Our findings suggest that more children in the household might destroy the relationships between parenting skills and cognitive development of children who have siblings, which is also detrimental to their future performances.

Last but not least, the findings in this paper suggest that, for one thing, different styles of parenting skills could bring different benefits to children, which is similar to the evidence from developed countries [10,11,12,13]; for another, the most beneficial parenting skill for rural children in China is low coercive parenting, followed by the parent–child relationship, positive encouragement, and parental adjustment, respectively, which provide much more information to caregivers in rural western China.

We acknowledge that our study has some limitations. First, our sample is not nationally representative, so the conclusions may not be simply generalized to all households in the whole country. Due to the lack of urban samples, we are unable to examine whether the relationship and the heterogeneity still hold for urban households, which have more resources to invest in childhood development. The impact of household resource constraints on the relationship between parenting skills and development outcomes is worthy of future investigations. We leave this issue for future research. Second, while the OLS estimates are helpful to understand the predictive ability of parenting skills on child development outcomes, the evidence does not state causal inference. Causal relationships between parenting skills and early childhood development call for further investigation as well.

## 5. Conclusions

Despite these limitations, the findings of this study still have important implications. First and foremost, considering the strong correlation between parenting skills and development outcomes, in particular, children’s social-emotional development, interventions aimed at improving caregivers’ parenting skills, might be necessary to foster human capital development in rural China. Second, considering the heterogenous predictive abilities of different dimensions of parenting skills, the interventions could be designed to improve parental consistency if the child’s cognitive development is the focus, while decreasing the caregiver’s coercive parenting could be more helpful to booster the children’s language, motor, and social-emotional development. Third, for rural children who have siblings and live in dibao households, other interventions to directly increase parental investments might be more effective. 

In some areas of rural China, such as Shaanxi Province [36], Yunnan and Hebei Province [37], some parenting intervention programs have been conducted to improve the child’s development outcomes. The findings in this paper indicate that the improvement in a caregiver’s parenting skills may be one important channel between parenting intervention and early childhood development. We believe that this is informative for the policymakers to design more targeted public policy to improve human capital formation in rural areas.

## Figures and Tables

**Table 1:** Descriptive statistics.

Variable	Definition	Mean	S. D.	Min	Max	N
Dependent variable						
*cog*	cognitive score	96.00	12.56	55	135	1715
*lang*	language score	92.41	13.47	50	135	1715
*motor*	motor score	97.21	16.57	46	148	1715
*soemo*	social-emotional score	85.94	15.29	55	145	1715
Independent variable						
*pafas*	total Parent and Family Adjustment Scale (PAFAS) score	61.95	7.63	37	84	1715
Covariates						
*male*	dummy, 1 = male	0.52	0.50	0	1	1715
*month*	child’s age in months	14.39	5.50	6	24	1715
*sib*	dummy, 1 = child has siblings	0.51	0.50	0	1	1715
*cage*	caregiver’s age	35.21	12.27	17	76	1715
*cedu*	caregiver’s year of schooling	8.07	3.33	0	16	1715
*dibao*	dummy, 1 = household receives dibao	0.11	0.31	0	1	1715

Data source: Authors’ survey.

**Table 2:** Association between parenting skills and development outcomes.

	Cognition	Language	Motor	Social-Emotion
	(1)	(2)	(3)	(4)
Pafas	0.08 ** (0.04)	0.16 *** (0.05)	0.11 ** (0.05)	0.48 *** (0.07)
Standardized coefficient of Pafas	0.05	0.09	0.05	0.24
Covariates	Yes	Yes	Yes	Yes
Village FE	Yes	Yes	Yes	Yes
Observations	1715	1715	1715	1715

Notes: (i) Ordinary least squares (OLS) estimators are reported in the table, and robust standard errors presented in parentheses are clustered at the village level; (ii) *** *p* < 0.01, ** *p* < 0.05.

**Table 3:** Association between parenting skills and development outcomes by subsamples.

	Male = 1	Male = 0	Dibao = 1	Dibao = 0	Sib = 1	Sib = 0
	(1)	(2)	(3)	(4)	(5)	(6)
Panel A. The dependent variable is the child’s cognitive score						
Pafas	0.05 (0.06)	0.10 *(0.06)	0.04(0.20)	0.08 ** (0.04)	0.04 (0.07)	0.10 * (0.06)
Standardized coefficient of Pafas	0.03	0.06	0.02	0.05	0.02	0.06
Other covariates	Yes	Yes	Yes	Yes	Yes	Yes
Village FE	Yes	Yes	Yes	Yes	Yes	Yes
Observations	891	824	188	1527	870	845
Panel B. The dependent variable is the child’s language score						
Pafas	0.11* (0.06)	0.20 *** (0.06)	0.05 (0.153)	0.16 *** (0.04)	0.20 *** (0.06)	0.14 ** (0.06)
Standardized coefficient of Pafas	0.06	0.11	0.03	0.09	0.11	0.08
Other covariates	Yes	Yes	Yes	Yes	Yes	Yes
Village FE	Yes	Yes	Yes	Yes	Yes	Yes
Observations	891	824	188	1527	870	845
Panel C. The dependent variable is the child’s motor score						
Pafas	0.09 (0.07)	0.11* (0.06)	0.34 (0.26)	0.10** (0.05)	0.08 (0.06)	0.11* (0.06)
Standardized coefficient of Pafas	0.04	0.05	0.16	0.05	0.03	0.06
Other covariates	Yes	Yes	Yes	Yes	Yes	Yes
Village FE	Yes	Yes	Yes	Yes	Yes	Yes
Observations	891	824	188	1527	870	845
Panel D. The dependent variable is the child’s social-emotional score						
Pafas	0.42*** (0.07)	0.53*** (0.07)	0.53*** (0.19)	0.48*** (0.05)	0.53*** (0.07)	0.47*** (0.07)
Standardized coefficient of Pafas	0.21	0.27	0.27	0.24	0.27	0.23
Other covariates	Yes	Yes	Yes	Yes	Yes	Yes
Village FE	Yes	Yes	Yes	Yes	Yes	Yes
Observations	891	824	188	1527	870	845

Notes: (i) OLS estimators are reported in the table, and robust standard errors presented in parentheses are clustered at the village level. (ii) *** *p* < 0.01, ** *p* < 0.05, * *p* < 0.1.

**Table 4:** Association between different dimensions of parenting skills and development outcomes.

	Cognition	Language	Motor	Social-Emotion
	(1)	(2)	(3)	(4)
*Consist*	0.38 ** (0.16)	0.05 (0.15)	0.22 (0.20)	0.31 (0.20)
*Coer*	0.21 (0.14)	0.35 ** (0.15)	0.49 *** (0.20)	0.33 * (0.17)
*Encour*	0.10 (0.19)	–0.21 (0.23)	0.24 (0.20)	1.13 *** (0.23)
*Rela_pc*	0.07 (0.15)	0.53 ^***^ (0.17)	0.09 (0.16)	1.00 *** (0.18)
*Adjust*	0.01 (0.15)	0.10 (0.14)	-0.05 (0.18)	0.54 *** (0.18)
*Rela_fmy*	–0.12 (0.26)	0.22 (0.21)	0.08 (0.25)	–0.04 (0.26)
*Team*	0.22 (0.23)	–0.28 (0.26)	0.30 (0.29)	0.30 (0.28)
Covariates	Yes	Yes	Yes	Yes
Village FE	Yes	Yes	Yes	Yes
Observations	1715	1715	1715	1715

Notes: (i) The independent variables are the PAFAS seven subscales—parental consistency (*consist*), coercive parenting (*coer*), positive encouragement (*encour*), parent–child relationship (*rela_pc*), parental adjustment (*adjust*), family relationships (*rela_fmy*), and parental teamwork (*team*). (ii) The OLS estimators are reported in the table, and robust standard errors presented in parentheses are clustered at the village level. (iii) *** *p* < 0.01, ** *p* < 0.05, * *p* < 0.1.

## Appendix A

**Table A1:** Parent and Family Adjustment Scales (PAFAS).

Subscale	Cronbach’s Alpha
**Parental consistency**	
1. If my child doesn’t do what they’re told to do, I give in and do it myself.	0.77
2. I follow through with a consequence (e.g. take away a toy) when my child misbehaves.	0.76
3. I threaten something (e.g. to turn off TV) when my child misbehaves but I don’t follow through.	0.76
4. I deal with my child’s misbehaviour the same way all the time.	0.77
5. I give my child what they want when they get angry or upset.	0.78
**Coercive parenting**	
1. I shout or get angry with my child when they misbehave.	0.75
2. I try to make my child feel bad (e.g. guilt or shame) for misbehaving to teach them a lesson.	0.77
3. I spank (smack) my child when they misbehave.	0.76
4. I argue with my child about their behaviour/attitude.	0.78
5. I get annoyed with my child.	0.77
**Positive encouragement**	
1. I give my child a treat, reward or fun activity for behaving well.	0.78
2. I praise my child when they behave well.	0.77
3. I give my child attention (e.g. a hug, wink, smile or kiss) when they behave well.	0.77
**Parent-child relationship**	
1. I chat/talk with my child.	0.76
2. I enjoy giving my child hugs, kisses and cuddles.	0.75
3. I am proud of my child.	0.75
4. I enjoy spending time with my child.	0.75
5. I have a good relationship with my child.	0.75
**Parental adjustment**	
1. I feel stressed or worried.	0.77
2. I feel happy.	0.75
3. I feel sad or depressed.	0.77
4. I feel satisfied with my life.	0.75
5. I cope with the emotional demands of being a parent.	0.76
**Family relationships**	
1. Our family members help or support each other.	0.75
2. Our family members get on well with each other.	0.75
3. Our family members fight or argue.	0.77
4. Our family members criticize or put each other down.	0.77
**Parental teamwork**	
1. I work as a team with my partner in parenting.	0.76
2. I disagree with my partner about parenting.	0.77
3. I have a good relationship with my partner.	0.76
**Total**	0.77

Notes: (i) You may find the practical version in Sanders et al. (2014); (ii) The rating scale is as follows: 0: Not true of me at all, 1: True of me a little, or some of the time, 2: True of me quite a lot, or a good part of the time, 3: True of me very much, or most of the time.
